# Associations among tourist camp management, high and low tourist seasons, and welfare factors in female Asian elephants in Thailand

**DOI:** 10.1371/journal.pone.0218579

**Published:** 2019-06-17

**Authors:** Treepradab Norkaew, Janine L. Brown, Chatchote Thitaram, Pakkanut Bansiddhi, Chaleamchat Somgird, Veerasak Punyapornwithaya, Khanittha Punturee, Preeyanat Vongchan, Nopphamas Somboon, Jaruwan Khonmee

**Affiliations:** 1 Center of Elephant and Wildlife Research, Faculty of Veterinary Medicine, Chiang Mai University, Chiang Mai, Thailand; 2 Center for Species Survival, Smithsonian Conservation Biology Institute, Front Royal, Virginia, United States of America; 3 Department of Companion Animal and Wildlife Clinic, Faculty of Veterinary Medicine, Chiang Mai University, Chiang Mai, Thailand; 4 Department of Food Animal Clinic, Faculty of Veterinary Medicine, Chiang Mai University, Chiang Mai, Thailand; 5 Department of Medical Technology, Faculty of Associated Medical Sciences, Chiang Mai University, Chiang Mai, Thailand; 6 Small Animal Teaching Hospital, Faculty of Veterinary Medicine, Chiang Mai University, Chiang Mai, Thailand; 7 Department of Veterinary Bioscience and Veterinary Public Health, Faculty of Veterinary Medicine, Chiang Mai University, Chiang Mai, Thailand; University of Tasmania, AUSTRALIA

## Abstract

This study investigated how camp management and tourist activities affect body condition, adrenocortical function, lipid profiles and metabolic status in female tourist elephants. We compared twice monthly serum insulin, glucose, fructosamine, total cholesterol (TC), triglyceride (TG), low density lipoprotein (LDL), high density lipoprotein (HDL), and fecal glucocorticoid metabolite (FGM) concentrations to body condition scores (BCS) at five camps with different management styles (e.g., tourist activities, work type, diet) between the High (November–February) and Low (March–October) tourist seasons. There were significant camp effects on health parameters, with BCS, TC, HDL, insulin and glucose being among the highest, and G:I being the lowest (less heathy) in elephants at an observation camp compared to those at camps where elephants received exercise by providing rides to tourists. Differences between High and Low tourist season months also were found for all measures, except TG and FGM concentrations. Both work time and walking distance were negatively correlated to glucose, fructosamine and insulin, while walking distance was negatively related to FGM concentrations. By contrast, positive associations were found between tourist number and BCS, TG, and insulin, perhaps related to tourists feeding elephants. Quantity of supplementary diet items (e.g., bananas, sugar cane, pumpkin) were positively correlated with FGM concentrations, glucose, fructosamine, and insulin. This study provides evidence that body condition, adrenal activity, metabolic markers, and lipid profiles in captive elephants may be affected by visitor numbers, work activities, and the amount of supplementary foods offered by tourists. Some activities appear to have negative (e.g., feeding), while others (e.g., exercise) may have positive effects on health and welfare. We conclude that camps adopting a more hands-off approach to tourism need to ensure elephants remain healthy by providing environments that encourage activity and rely on more natural diets or foraging.

## Introduction

Elephants had been used in the logging industry in Thailand for centuries; however, in 1989 a logging ban was initiated by the government that left thousands of elephants and their mahouts unemployed, and brought on the use of privately owned animals in tourism. Elephants today play an important role in the economy of Thailand, with about 3,500 mostly (95%) privately owned (Asian Elephant Specialist Group, 2017) [1] being used primarily for tourism. Over the past two decades, the number of elephant camps has increased significantly, especially in the northern region, which consists of 40% of camps in the country, mainly in Chiang Mai (37% of the country) (National Institute of Elephant Research and Health Service, Lampang, Thailand). Elephants participate in a variety of activities, including shows, riding, feeding, and bathing by tourists. In recent years, the welfare of captive elephants has become a topic of intense debate among government agencies, animal rights groups, scientists, and the general public. Management differs across elephant camps with respect to tourist numbers, intensity of activities, nutrition, breeding, restraint, and control, all of which can have impacts on health, behavior and welfare. Tourist activities have been shown to compromise welfare and negatively affect behavior and physiology in other species, resulting in increased hiding behaviors [2, 3], heightened vigilance [4, 5], stereotypies [6, 7], poor body condition [8], and elevated glucocorticoid (GC) levels [9–11]. Most studies of this type have been conducted in western zoo animals, with few focusing on captive animals in range countries, including elephants.

One of the most popular tourist activities is feeding elephants treats like bananas and sugar cane, although there is a minimal understanding of the potential health impacts. Feeding of unnatural or unhealthy high energy food items can compromise health and fitness [12], resulting in obesity, metabolic syndromes, and energy imbalances [13, 14]. Elephant riding, with saddles or bareback, is another popular activity enjoyed by tourists, although it has come under intense criticism in recent years, with TripAdvisor no longer selling tickets to venues where elephant rides are offered. However, in a recent study, the number of working hours related primarily to giving rides was related to lower adrenal activity, based on fecal glucocorticoid metabolite (FGM) measures and better body condition in elephants using camp-based information [15], and so this activity may have positive effects on health parameters. In other species, however, high numbers of work hours have been associated with increased adrenal corticoid output [16, 17], and a number of negative health effects, such as poor body condition [18]. However, others report positive health effects of work [19], so relationships between work, health and well-being are not always clear [20], and likely depend on the type of work being done and how the work is perceived by the individual animal.

Assessing the impact of tourist activities on health and well-being can involve measures of stress hormones and how altered adrenal activity affects metabolic function and lipid parameters. Glucocorticoids modulate a number of physiological actions involved in metabolic, inflammatory, cardiovascular, and behavioral processes. Mechanisms by which GCs coordinate these effects include increasing glucose production from hepatic cells [21], decreasing glucose uptake into muscle and adipose tissue [22, 23], increasing lipolysis [24], and decreasing insulin release from pancreatic cells [25]. Working elephants in Thailand interact with the public in a variety of ways. Often these activities are not closely monitored or regulated, and could be sources of stress to individual animals. Recently, relationships were found between adrenal and metabolic hormones and lipid profiles in Asian elephants, with positive correlations between FGM and total cholesterol (TC), high density lipoprotein (HDL), glucose, and insulin, and negative correlations with the glucose-to-insulin (G:I) ratio [15]. Because it is generally not possible to fast elephants before blood sample collection in tourist camps, and elephants have access to forage overnight, a G:I ratio is calculated, which detects insulin sensitivity in women [26], with lower values reflecting metabolic abnormalities. Serum fructosamine is often measured, which reflects glucose levels over the previous 2–3 weeks, and can be used to monitor and control blood sugar concentrations in unfasted subjects, including diabetic patients [27], dogs and cats [28] and elephants [15]. It has been reported to have positive correlations with body weight [29], body mass index [30], and waist circumference [31] in other species. The present study built on prior research [15] to examine camp differences and how tourist numbers and working hours affect adrenal, lipid and metabolic function in captive elephants in northern Thailand, and if there are any differences in physiological function between the High (November–February) and Low (March–October) tourist seasons in the region. This study also expanded on evaluating how the feeding of high calorie treats by tourists impacts these health and welfare biomarkers.

## Materials and methods

### Animals

This study was approved by the Faculty of Veterinary Medicine, Chiang Mai University, Animal Care and Use Committee (FVM-ACUC; permit number S39/2559). Thirty-three adult female Asian elephants (age range, 18–50; mean, 34.2 ± 7.3 years) were housed at five tourist camps within 43–72 km of the Chiang Mai University Veterinary Faculty (latitude 18°47'N, longitude 98°59'E, altitude 330 m) (Table 1). At four of the camps, tourists interacted with elephants through riding programs (bareback or with a saddle) and feeding of supplementary foods. The fifth camp offered no tourist activities other than observation of elephants in a large field, feeding of supplementary foods by mahouts, and being taken for a bath at a local river. Work time per day was the time elephants were actively involved in tourist activities. For saddle and bareback riding, it equated to the number of rounds of riding per day times the minutes per riding round. Walking distance was the distance elephants were walked per day. Elephants were fed primarily corn stalk, Napier grass (*Pennisetum purpureum*) and bana grass (*Pennisetum purpureum* X, *P*. *americanum* hybrid) with limited access to fresh water. Supplementary foods consisted of bamboo, sugarcane, bananas, pumpkins, watermelon and cucumber that were primarily offered by tourists. Animals were given an annual physical examination by staff veterinarians, and were in good health during the study.

**Table 1:** Description of the elephant camps and elephants that participated in the study. Data were averaged over the 1-year study period during the High and Low tourist seasons and shown as mean ± standard error of the mean (SEM). Information includes the number of years the camp has been in operation (camp age), the total number of elephants in the camp, the number of female elephants participating in the study, participating elephant mean age and range, type of work with tourists, work time and walking distance per day, and primary and supplemental food items.

Variable	Camp A	Camp B	Camp C	Camp D	Camp E
Camp age (years)	9	27	29	14	40
Total elephants in camp	46	66	52	68	76
Total participating elephants	6	6	6	11	4
Elephant age (years)	28.50 ± 1.80	36.80 ± 2.90	35.30 ± 3.80	35.80 ± 2.30	32.20 ± 3.00
	(22–34)	(23–43)	(20–45)	(25–50)	(22–40)
Type of work	Bareback riding	Saddle riding	Saddle riding	No riding	Saddle riding
Total work time (min/day)	66.70 ± 1.58	180.00 ± 7.11	204.00 ± 7.94	0	236.00 ± 8.42
- number of round	1.67 ± 0.04	8.99 ± 0.36	5.11 ± 0.20	0	9.33 ± 0.35
- round time (min/round)	40	20	40	0	20
Total walking distance (km/day)	1.63 ± 0.04	4.48 ± 0.18	5.05 ± 0.21	0	4.77 ± 0.17
Diet					
Primary	Napier grass, cornstalk	Napier grass, cornstalk	Napier grass, cornstalk	Napier grass, bana grass, cornstalk	Napier grass
- Amount per day (kg/day)	150 ± 0.00	168 ± 1.70	100 ± 0.00	165 ± 0.00	168 ± 4.70
Supplementary	Bamboo, sugarcane, banana	Banana, sugarcane	Banana, sugarcane	Hay, banana, watermelon, pumpkin, cucumber	Bamboo, sugarcane, banana
- Amount per day (kg/day)	30	10	10	20	5

### Sample collection

Blood samples (10 ml) were collected from an ear vein by elephant camp staff or Chiang Mai University veterinarians twice monthly for 1 year. All elephants were conditioned to the blood sampling procedure. Blood was centrifuged at 1,500 x g for 10 minutes within a few hours of collection, and the serum stored at -20°C until analysis. Fecal samples were collected at the same time. Immediately upon defecation, the dung bolus was mixed and several subsamples collected (~50 g/sample). Samples were stored on ice in a styrofoam cooler, transported to CMU and then frozen at -20°C until processing and analysis. Total blood and fecal samples collected were 264 during High season and 528 during the Low season.

### Body condition scoring

Once every 2 months, rear and side view photographs were taken to create a body condition score (BCS) for each elephant. Photos permitted a visual evaluation of the backbone, rib and pelvic bone areas, and body condition was scored on a scale of 1–5 (1 = thinnest; 5 = fattest) as described by Morfeld *et al*. [32], except that scoring was done in 0.5-point, rather than 1-point, increments. All photos were evaluated by three experienced elephant veterinarians, and the scores averaged. Intra-class correlations determined the inter-assessor reliability was 0.85.

### Metabolic marker analyses

Serum glucose was measured by a hexokinase method using an automated glucose analyzer (Glucinet T01-149, Bayer, Barcelona, Spain), with quinoneimine measured at 530 nm. Serum fructosamine was measured by a colorimetric method using nitrobluetetrazolium [33] in a Biosystems BA400 clinical chemistry analyzer (Biosystems S.A., Barcelona, Spain). A solid-phase, two-site bovine insulin enzyme immunoassay (EIA; Cat. No. 10-1113-01; Mercodia, Uppsala, Sweden), validated for elephants, was used to measure serum insulin concentrations [32]. Colorimetric responses were determined spectrophotometrically at 450 nm filter with an Opsys MR Microplate Reader (TECAN Sunrise microplate reader; Salzburg, Austria). All samples were analyzed in duplicate; intra- and inter-assay CVs were <10% and <15%, respectively.

### Lipid profile analysis

Serum lipids were quantified using a Mindray BS Series analyzer (Mindray BS-380, Shenzhen Mindray Bio-Medical Electronics Co., Ltd.). Total cholesterol was measured by a cholesterol oxidase-peroxidase (CHOD-POD) method. Triglycerides were measured by a glycerokinase peroxidase-peroxidase (GPO-POD) method, with a sensitivity of 0.1 mmol/l (99.7% confidence). The lowest measurable concentration was 0.1 mmol/l (99.7% confidence) for TC, and 0.05 mmol/l for both HDL and LDL.

### Steroid extraction and GC metabolite analysis

The fecal extraction technique is described in *Norkaew et al*. [15]. Briefly, wet samples were dried in a conventional oven at 60°C for ~24–48 hours and stored at -20°C until extraction. Frozen dried fecal samples were thawed at room temperature (RT), mixed well and 0.1 g (± 0.01) of dry powdered feces extracted twice in 90% ethanol in distilled water by boiling in a water bath (96°C) for 20 minutes and adding 100% ethanol as needed to keep from boiling dry. Samples were centrifuged at 1,500 x g for 20 min, and the combined supernatants dried under air in a 50°C water bath. Dried extracts were reconstituted in methanol and diluted 1:3 in assay buffer (Cat. No. X065, Arbor Assays, Arbor, MI, USA) and stored at –20°C until enzyme immunoassay (EIA) analysis.

Concentrations of FGM were determined using a double-antibody enzyme EIA with a polyclonal rabbit anti-corticosterone antibody (CJM006) validated for Asian elephants [34] and described by *Norkaew et al*. [15]. Assay sensitivity (based on 90% binding) was 0.14 ng/ml (0.014 ng/g). Samples were analyzed in duplicate; intra- and inter-assay CVs were <10% and <15%, respectively. Fecal data are expressed as ‘ng/g’ of dried feces.

### Statistical analysis

Descriptive data were reported as mean ± standard error of the mean (SEM) and Camp management variables were presented as a range or a frequency, depending on the type of data. Statistical analyses were performed using R version 3.4.0 [35]. Repeated measures data were analyzed using Generalized Estimating Equations (GEE) to determine how BCS, FGM, metabolic and lipid panel results were affected by tourist numbers, work time, and walking distance. High (November–February) and Low (March–October) tourist seasons were defined by the Tourism Authority of Thailand. Effect of individual elephant was included in the GEE analysis. Differences in mean FGM, metabolic (insulin, glucose and fructosamine), lipid (TC, TG, LDL and HDL) concentrations and work type between High and Low tourist seasons and among camps were analyzed by Tukey’s post-hoc tests using a P value correction. Correlations between diet and BCS, FGM and metabolic hormones or lipid measures were analyzed using linear regression tests for aggregated data. Differences in mean BCS, FGM, metabolic hormone and lipid measures between or within camp in High and Low tourist seasons were analyzed using Tukey’s post-hoc tests. The significance level was set at α = 0.05.

## Results

There was notable variation across the camps in work activities for elephants, with bareback riding for Camp A, saddle riding for Camps B, C and E, and no riding for Camp D. Camp C had the highest walking per day and Camp A had the lowest. Camp D elephants did not work at all; tourists watched them at a close distance in a field (Table 1). There also were significant differences across camps in adrenal activity, metabolic maker and lipid profiles (Table 2), with BCS, TC, HDL, insulin and glucose being among the highest, and G:I being among the lowest in Camp D, the facility with no work activities for elephants. Glucose and insulin concentrations in elephants at Camp A also were high, and during the High season, the G:I in that camp was the lowest. Variability among elephants in concentrations of FGM was high and shown in Supplementary Table 1. In addition to metabolic factors, FGM concentrations in elephants at Camp D also were high, similar to Camp A, while those in Camps C and E were the lowest. Camp E had the highest TC and TG values, while Camps C and E had the lowest HDL, and Camp E had the highest LDL concentrations.

**Table 2:** Differences in FGM, metabolic and lipid measures in captive elephants under different management conditions. Mean (± SEM) and range values (min–max) are presented. Fecal samples were collected for glucocorticoid analyses, and visual body condition scores were determined based on a set of photographs. Blood samples were collected to assess lipid and metabolic status for 1 year including High and Low season. The elephants were housed at five elephant camps in Northern Thailand, and studied to determine how management (e.g., work activities, feeding, work type) affected physiological function.

Factors	Camp A	Camp B	Camp C	Camp D	Camp E
FGM (ng/g)	60.40 ± 2.43^c^	49.60 ± 2.40^b^	39.10 ± 1.41^a^	58.20 ± 1.75^c^	39.60 ± 2.06^a^
	(20.00–142.00)	(11.90–194.00)	(15.40–153.00)	(16.60–173.00)	(12.40–110.00)
BCS	3.25 ± 0.06^a^	3.44 ± 0.08^a^	3.21 ± 0.09^a^	4.17 ± 0.10^b^	3.43 ± 0.10^a^
	(3.00–4.00)	(3.00–4.50)	(2.00–4.00)	(3.00–5.00)	(3.00–4.00)
TC (mg/dL)	35.50± 0.88^a^	35.50 ± 0.72^a^	34.60 ± 0.82^a^	39.5 ± 0.50^b^	39.70 ± 0.74^b^
	(10.00–76.00)	(22.00–76.00)	(20.00–109.00)	(21.00–77.00)	(28.00–72.00)
TG (mg/dL)	25.50 ± 1.36^a^	29.40 ± 1.62^a^	25.50 ± 1.33^a^	28.80 ± 1.27^a^	36.20 ± 1.74^b^
	(5.00–88.00)	(8.00–113.00)	(4.00–94.00)	(6.00–157.00)	(11.00–110.00)
HDL (mg/dL)	11.40 ± 0.21^b^	11.40 ± 0.17^b^	10.30 ± 0.14^a^	13.00 ± 0.20^c^	10.70 ± 0.31^a^^b^
	(3.00–18.00)	(7.00–19.00)	(2.00–15.00)	(7.00–26.00)	(3.00–26.00)
LDL (mg/dL)	26.70 ± 0.94^a^^b^	25.00 ± 0.66^a^	26.00 ± 0.91^a^	29.50 ± 0.43^b^^c^	30.40 ± 0.60^c^
	(11.00–53.00)	(9.00–51.00)	(8.00–107.00)	(15.00–67.00)	(19.00–47.00)
Glucose (mg/dL)	96.40 ± 2.27^b^	78.20 ± 1.23^a^	78.50 ± 1.43^a^	100.90 ± 1.29^b^	79.60 ± 1.47^a^
	(55.00–172.00)	(52.00–125.00)	(50.00–160.00)	(52.00–180.00)	(55.00–118.00)
Fructosamine (mM)	0.60 ± 0.004^b^^c^	0.59 ± 0.006^b^	0.57 ± 0.005^a^	0.61± 0.003^c^	0.56 ± 0.006^a^
	(0.50–0.77)	(0.45–0.92)	(0.47–0.86)	(0.39–0.78)	(0.38–0.70)
Insulin (ng/ml)	0.99 ± 0.16^c^^d^	0.45 ± 0.05^a^^b^	0.34 ± 0.04^a^	1.08 ± 0.08^d^	0.66 ± 0.09^b^^c^
	(0.03–5.91)	(0.03–1.77)	(0.03–1.98)	(0.02–3.93)	(0.08–2.83)
G:I	171.00 ± 29.20^a^^b^	240.00 ± 21.92^b^	238.00 ± 22.17^b^	141.00 ± 8.77^a^	181.00 ± 16.23^a^^b^
	(22.70–722.00)	(58.30–712.00)	(47.90–660.00)	(23.70–569.00)	(37.50–446.00)

^**a,b,c**^Row values for each Factor differ significantly across the five Camps (P<0.05).

FGM = fecal glucocorticoid metabolites; BCS = body condition score; TC = total cholesterol; TG = triglycerides

HDL = high density lipoproteins; LDL = low density lipoproteins; G:I = glucose to insulin ratio.

Overall, during the High tourist season, elephants exhibited higher FGM, TC, glucose and insulin concentrations than during the Low season (Table 3). In particular, insulin concentrations were 44% higher during the High compared to the Low season. Although glucose also was higher, the G:I did not differ between seasons. Conversely, fructosamine was higher in the Low season. Elephant work time and walking distance in the High season were nearly double those in the Low season, except camp D where, although camp operating hours were unchanged, elephants did not participate in riding activities.

**Table 3:** Differences in FGM, metabolic and lipid measures in captive elephants evaluated during the High and Low tourist season. Mean (± SEM) and range values (min–max) are presented. Fecal samples were collected for glucocorticoid analyses, and visual body condition scores were determined based on a set of photographs. Blood samples were collected to assess lipid and metabolic status. The elephants were housed at five elephant camps in Northern Thailand with different tourist activities.

Factors	High season	Low season
FGM (ng/g)	58.12 ± 2.24^b^	48.33 ± 1.03^a^
	(19.59–194.17)	(11.42–147.07)
BCS	3.62 ± 0.07	3.46 ± 0.08
	(2.00–5.00)	(2.00–5.00)
TC (mg/dL)	38.44 ± 0.58^b^	36.83 ± 0.39^a^
	(22.00–109.00)	(10.00–85.00)
TG (mg/dL)	28.00 ± 1.11	29.00 ± 0.82
	(7.00–110.00)	(4.00–157.00)
HDL (mg/dL)	11.90 ± 0.18	11.50 ± 0.12
	(3.00–26.00)	(2.00–26.00)
LDL (mg/dL)	28.70 ± 0.50	27.30 ± 0.40
	(9.00–89.00)	(8.00–107.00)
Glucose (mg/dL)	92.40 ± 1.53^b^	87.20 ± 0.88^a^
	(52.00–172.00)	(50.00–180.00)
Fructosamine (mM)	0.58 ± 0.01^a^	0.60 ± 0.01^b^
	(0.45–0.77)	(0.38–0.92)
Insulin (ng/ml)	0.94 ± 0.09^b^	0.65 ± 0.04^a^
	(0.03–5.91)	(0.02–3.37)
G:I	163.00 ± 12.40	195.00 ± 10.50
	(22.70–613.00)	(34.40–722.00)
Operation time (hour/day)	6.38 ± 0.05	6.38 ± 0.03
	(5.00–7.00)	(5.00–7.00)
Work time (hr/day)^1^	3.83 ± 0.12^b^	2.22 ± 0.05^a^
	(0.00–400.00)	(0.00–400.00)
Walking distance (km/day)^1^	5.52 ± 0.20^b^	3.11 ± 0.87^a^
	(0.00–10.00)	(0.00–10.00)

^**a,b**^Row values for each Factor differ significantly between the High and Low tourist seasons (P<0.05).

^1^Excludes Camp D where elephants did not participate in riding activities.

FGM = fecal glucocorticoid metabolites; TC = total cholesterol; TG = triglycerides; HDL = high density lipoproteins; LDL = low density lipoproteins; G:I = glucose to insulin ratio.

High: November–February, Low: March–October (According to the Tourism Authority of Thailand).

Table 4 shows differences among the five camps in FGM, metabolic and lipid measures between High and Low tourist seasons within each camp. The results showed that all camps except Camp E had higher FGM concentrations (18–30%) during the High than the Low tourist season. Camps A and D had the highest, while Camps C and E had the lowest FGM concentrations in both seasons. During the High tourist season, Camps A and D had higher FGM concentrations than Camps C and E. Similarly, during the Low tourist season, Camp A had higher FGM concentrations than Camps B, C and E, while Camp D had higher FGM concentrations than Camps C and E. For BCS, the only camp with a seasonal difference was Camp A, with lower condition in the Low season. Camp D had the highest BCS in both High and Low tourist seasons.

**Table 4:** Differences in FGM, metabolic and lipid measures in captive elephants managed at five tourist camps. Fecal samples were collected for glucocorticoid analyses, and visual body condition scores were determined based on a set of photographs. The elephants were housed at five elephant camps in Northern Thailand, and studied to determine if differences in management (e.g., work activities, feeding, work type) affected physiological function.

Factor		Camp A	Camp B	Camp C	Camp D	Camp E
FGM (ng/g)	High	70.40 ± 3.86^c^*	59.70 ± 4.73^b^^c^*	44.70 ± 3.10^a^^b^*	64.70 ± 2.89^c^*	40.40 ± 2.21^a^
	Low	59.70 ± 2.52^d^	46.20 ±2.47^b^^c^	36.40 ± 1.14^a^	54.30 ± 1.17^c^^d^	39.40 ± 2.58^a^^b^
BCS	High	3.36 ± 0.05^a^*	3.59 ± 0.08^a^^b^	3.38 ± 0.07^a^	4.08 ± 0.08^b^	3.25 ± 0.09^a^
	Low	3.10 ± 0.02^a^	3.37 ± 0.05^a^^b^	3.04 ± 0.04^a^	3.91 ± 0.06^b^	3.50 ± 0.05^b^
TC (mg/dL)	High	38.90 ± 1.60^a^^b^	35.90 ± 1.19^a^	37.40 ± 1.78^a^^b^*	40.20 ± 0.75^b^	38.60 ± 0.83^a^^b^
	Low	35.60 ± 0.95^a^	35.30 ± 0.89^a^	33.30 ± 0.82^a^	39.20 ± 0.62^b^	40.20 ± 0.99^b^
TG (mg/dL)	High	25.30 ± 2.23^a^	27.60 ± 2.14^a^	26.70 ± 2.59^a^	26.90 ± 1.86^a^	36.80 ± 3.52^a^
	Low	25.50 ± 1.27^a^	30.30 ± 2.15^a^^b^	24.80 ± 1.52^a^	29.80 ± 1.59^a^^b^	35.80 ± 1.85^b^
HDL (mg/dL)	High	12.30 ± 0.32^b^^c^*	11.60 ± 0.35^b^	10.10 ± 0.23^a^	13.10 ± 0.29^c^	11.30 ± 0.73^a^^b^^c^
	Low	11.00 ± 0.21^a^^b^	11.30 ± 0.19^b^	10.30 ± 0.17^a^	12.90 ± 0.25^c^	10.40 ± 0.23^a^
LDL (mg/dL)	High	29.10 ± 1.26^a^^b^	25.70 ± 1.16^a^	28.10 ± 1.48^a^^b^	30.00 ± 0.63^b^	30.10 ± 0.84^b^
	Low	26.50 ± 0.92^a^	24.70 ± 0.73^a^	24.90 ± 1.06^a^	29.20 ± 0.52^b^	30.60 ± 0.74^b^
Glucose (mg/dL)	High	104.00 ± 3.60^b^*	77.80 ± 2.07^a^	78.20 ± 2.53^a^	108.00 ± 2.28^b^*	81.50 ± 2.98^a^
	Low	91.50 ± 2.08^b^	78.40 ± 1.46^a^	78.60 ± .74^a^	97.60 ± 1.44^b^	78.60 ± 1.56^a^
Fructosamine (mM)	High	0.60 ± 0.005^c^	0.57 ± 0.007^b^*	0.54 ± 0.005^a^*	0.60 ± 0.005^c^	0.55 ± 0.006^a^^b^
	Low	0.61 ± 0.005^b^^c^	0.60 ± 0.008^b^^c^	0.58 ± 0.0007^a^^b^	0.61 ± 0.004^c^	0.56 ± 0.008^a^
Insulin (ng/ml)	High	1.52 ± 0.23^b^*	0.51 ± 0.06^a^	0.32 ± 0.05^a^	1.36 ± 0.10^b^*	0.73 ± 0.11^a^^b^
	Low	0.69 ± 0.07^a^^b^	0.42 ± 0.04^a^	0.36 ± 0.04^a^	0.95 ± 0.06^b^	0.63 ± 0.07^a^^b^
G:I	High	82.30 ± 9.74^a^*	214.00 ± 17.20^b^	274.00 ± 24.00^b^	119.00 ± 8.30^a^	174.00 ± 20.20^a^^b^
	Low	216.00 ± 21.30^a^^b^	254.00 ± 19.00^b^	221.00 ± 14.00^a^^b^	152.00 ± 7.47^a^	185.00 ± 12.40^a^^b^

^**a,b,c**^Row values for each Factor differ significantly between the camps.

*Indicates significant differences between High and Low seasons within the same camp (P<0.05).

FGM = fecal glucocorticoid metabolites; BCS = body condition score; TC = total cholesterol; TG = triglycerides; HDL = high density lipoproteins; LDL = low density lipoproteins; G:I = glucose to insulin ratio.

The only tourist season differences were TC in Camp C and HDL in Camp A, where concentrations were higher in the High season. Across camps, Camp D had higher HDL than Camps B and C during the High season, and the highest of all camps during Low season. For LDL, Camps D and E had higher LDL than Camp B during the High season, and Camps D and E had higher LDL than other camps during the Low season. Glucose and insulin concentrations in Camps A and D were 14% and 11% higher during the High than the Low tourist season, respectively. During the High season, glucose concentrations in Camp A were ~30% higher than in Camps B, C and E, and 16% higher than in Camps B, C and E. Glucose concentrations in Camp D were ~32–40% higher in Camps B, C and E during the High season and 24% higher than Camps B, C and E during the Low season. Moreover, insulin concentrations in Camps A and D during the High season were greater than all other camps. During the High season, insulin concentrations in Camp A were three, five and two times higher than Camps B, C and E, respectively. However, during the Low season, Camp D had higher insulin concentrations than Camps B and C. Because of the high insulin concentrations, Camp A had a low G:I during the High tourist season, 2.5 times than during the Low season. During the High tourist season, the G:Is in Camps A and D were significantly lower than in Camps B and C. During the Low tourist season, only Camp D had higher insulin concentrations than Camps B and C. There were significant tourist season differences between fructosamine concentrations in Camps B and C.

Relationships between tourist numbers and metabolic marker and lipid profiles were determined using GEE (Table 5). There were significant positive associations between tourist number and BCS, TG, and insulin. By contrast, tourist number were negatively correlated to glucose. Relationships between work time and walking distance on metabolic marker and lipid profiles are presented in Table 6, and show significant positive associations between work time and TC and TG. Walking distance also was positively related to TC. Both work time and walking distance were negatively correlated to glucose, fructosamine and insulin. Only walking distance was negatively related to FGM concentrations.

**Table 5:** Relationships between tourist number and health factors and adrenal steroid activity in captive Asian elephants in Thailand. Fecal samples were collected for FGM analyses, and visual body condition scores were determined based on a set of photographs. Blood samples were collected to assess lipid and metabolic status. Data were analyzed by GEE to determine the effect of tourist numbers on physiological function of 33 elephants housed at five elephant camps in Northern Thailand.

Factors	Tourist number		
	Intercept	Beta (x10^-5^)	P value
FGM (ng/g)	52.05	-27.90	0.210
BSC	3.19	0.91	0.017
TC (mg/dL)	35.40	6.87	0.140
TG (mg/dL)	25.50	21.50	0.002
HDL (mg/dL)	10.70	1.28	0.280
LDL (mg/dL)	27.10	-0.75	0.870
Glucose (mg/dL)	88.70	-41.80	<0.001
Fructosamine (mM)	0.59	-0.04	0.320
Insulin (ng/ml)	0.72	0.93	0.034
G:I	200.00	75.30	0.470

FGM = fecal glucocorticoid metabolites; BCS = body condition score; TC = total cholesterol; TG = triglycerides; HDL = high density lipoproteins; LDL = low density lipoproteins; G:I = glucose to insulin ratio.

**Table 6:** Relationships between work time and walking distance and health factors and adrenal steroid activity in captive Asian elephants in Thailand. Fecal samples were collected for glucocorticoid analyses, and visual body condition scores were determined based on a set of photographs. Blood samples were collected to assess lipid and metabolic status. Data were analyzed by GEE to determine the effect of work activities on physiological function of 33 elephants housed at five elephant camps in Northern Thailand.

Factors	Work time (hr/day)			Walking distance (km/day)		
	Intercept	Beta (x10^-2^)	P value	Intercept	Beta (x10^-4^)	P value
FGM (ng/g)	51.48	-2.000	0.140	53.73	-13.460	0.015
BCS	3.31	0.018	0.660	3.30	0.106	0.540
TC (mg/dL)	33.82	1.570	0.003	34.40	4.820	0.032
TG (mg/dL)	26.08	1.564	0.032	27.20	3.350	0.250
HDL (mg/dL)	10.74	0.125	0.320	10.80	0.241	0.590
LDL (mg/dL)	25.97	0.574	0.250	26.50	0.925	0.650
Glucose (mg/dL)	87.23	-2.826	0.003	88.60	-14.480	<0.001
Fructosamine (mM)	0.61	-0.016	<0.001	0.61	-0.063	<0.001
Insulin (ng/ml)	0.73	-0.089	0.039	0.81	-0.532	0.002
G:I	204.73	4.230	0.720	188.00	58.200	0.300

FGM = fecal glucocorticoid metabolites; BCS = body condition score; TC = total cholesterol; TG = triglycerides; HDL = high density lipoproteins; LDL = low density lipoproteins; G:I = glucose to insulin ratio.

In separate linear regression analyses of individual means (n = 33), the amount of primary diet was correlated to FGM, BCS, and insulin concentrations (Table 7), whereas the amount of supplementary diet was positively correlated with FGM, glucose, fructosamine, and insulin concentrations.

**Table 7:** Relationships between primary and supplementary diets and health factors and adrenal steroid activity in captive Asian elephants in Thailand. Fecal samples were collected for glucocorticoid analyses, and visual body condition scores were determined based on a set of photographs. Blood samples were collected to assess lipid and metabolic status. Data were analyzed by GEE to determine the effect of amounts of various dietary items on health and welfare of 33 elephants housed at five elephant camps in Northern Thailand.

Factors	Primary diet			Supplementary diet		
	Intercept	Beta	P value	Intercept	Beta	P value
FGM (ng/g)	32.64	0.126	0.037	35.62	0.986	<0.001
BCS	2.67	0.006	0.050	3.43	0.011	0.377
TC (mg/dL)	32.60	0.033	0.313	36.30	0.077	0.525
TG (mg/dL)	21.01	0.051	0.157	32.11	-0.214	0.104
HDL (mg/dL)	9.19	0.016	0.149	10.59	0.002	0.130
LDL (mg/dL)	24.64	0.031	0.463	26.70	0.084	0.464
Glucose (mg/dL)	72.11	0.111	0.119	73.12	0.967	<0.001
Fructosamine (mM)	0.542	0.001	0.081	0.563	0.002	0.008
Insulin (ng/ml)	-0.031	0.005	0.047	0.33	0.026	0.006
G:I	281.38	-0.582	0.258	215.92	-1.382	0.472

FGM = fecal glucocorticoid metabolites; BCS = body condition score; TC = total cholesterol; TG = triglycerides; HDL = high density lipoproteins; LDL = low density lipoproteins; G:I = glucose to insulin ratio.

## Discussion

This is the first study to examine metabolic and lipid parameters in Asian elephants under human care in Thailand across camps in relation to walking distance and working time, and provisioning of supplementary diet items, like bananas and sugar cane, by tourists during the High and Low tourist seasons. Significant differences across camps in FGM concentrations and metabolic status highlight the effect of tourist activities, and how supplemental feeding and lack of exercise may have negative consequences for health. There also was a significant tourist season effect on health status, with levels of several metabolic markers being higher during the High season, potentially reflecting higher numbers of tourists and associated activities. Higher numbers of tourists likely are associated with increases in amounts of food treats offered to elephants, given that feeding is one of the most popular tourist activities. However, samples were collected for one year from five camps, tourist number and management factor could there be variation from year to year, increasing of period of collection and sample sizes are essential to complete the better understanding of elephant’s health affecting factors.

The type and amount of supplementary food given to elephants varied with each camp, but generally consisted of items with a high sugar content and glycemic index, including bananas (glycemic index = 47), sugarcane (50), watermelon (72), and pumpkin (51; only camp D) (Sydney University's Glycemic Research Service). The glycemic index quantifies the widely variable increases in blood glucose after ingestion of different carbohydrates, with larger values associated with the development of metabolic disease [36]. High glycemic index foods induce an exaggerated insulin response, which can increase body fat and weight, and lead to insulin resistance, and eventual exhaustion of endocrine pancreatic function and insulin release [37, 38]. There is growing recognition and concern that obesity and metabolic conditions are negatively impacting the health of many species, including humans, companion and domestic animals. A similar health concern exists for zoo-held species, including elephants, that often are fed diets high in calories and given inadequate exercise [39–44]. Comparatively, the overall G:I average value for this study (G:I = 196) was slightly better than that in the U.S. (G:I = 110) [43], except Camp A (G:I = 82). Moreover, the overall glucose concentration in our study (glucose = 88.90) was slightly lower than that in the U.S. (glucose = 101.00) [43]. Elephants at two camps in particular, A and D, exhibited glucose and insulin concentrations that were higher overall compared to the other camps, and 1–2 times greater during the High tourist season. BCS at Camp A also was higher during the High season, while the G:I was the lowest during that time, indicative of metabolic derangements [43, 44]. These effects appear to be related, in part, to the feeding of greater amounts of supplementary foods at those two camps.

Fecal glucocorticoid metabolite concentrations also differed among camps, with A and D again being different in having the highest concentrations compared to the other camps. One possible explanation is that elephants in Camps A and D received less exercise than those in the other camps. Exercise has been shown to reduce stress, anxiety, and depression in a number of species [45], and can counter many of the physiological decrements of aging, and reduce risks for diseases linked to chronic elevations in cortisol [46]. Results of this study suggest that greater walking distances per day in tourist camp elephants may have a positive effect not only on BCS, but adrenal status as well. Thus, Camps A and D appear to operate in a way that results in overall higher glucocorticoid concentrations and poorer metabolic health. These camps were notably different in the activities elephants were exposed to. In Camp A, the main activity was bareback riding, but interestingly, in Camp D, tourists merely watched elephants in close proximity. What these two camps have in common are fewer hours of exercise (0–67 versus ≥180 hours/d) and higher amounts of supplementary items being fed (20–30 versus 5–10 kg/day). Thus, these results suggest that if elephants are not to be used for riding or other forms of physical exercise, then care must be taken not to overfeed them, and to limit the amounts of high calorie treats in particular.

Across camps, there was no relationship between tourist numbers and FGM concentrations, which agrees with studies suggesting that not all animals perceive human presence as a stressor [47, 48], although other studies have linked tourism-related activities to higher glucocorticoids in visited areas [7, 49, 50]. One explanation for the High tourist season effect on FGM concentrations is that elephants engage in more activity, and so are exposed to more stimuli that may increase adrenal glucocorticoid output.

## Conclusion

Using a generalized estimating equation (GEE) method, we found differences in concentrations of metabolic factors, lipid profiles and FGM concentrations across camps with different management styles, and between High and Low tourist seasons. Results suggest nutrition, work activities and tourist numbers may affect metabolic, lipid panel and FGM concentrations. We conclude that elephant well-being can be promoted by limiting the amount of high calorie treats given by tourists, ensuring animals receive appropriate amounts of exercise to reduce fat and increase muscle mass, and reducing stress by moderating the numbers of tourists interacting with individual elephants, especially during the High season.

Comparing FGM measures to elephants in U.S. zoos (mean, 124.69 ± 4.26 ng/g; range, 59.69–282.88 ng/g; n = 106) [Brown, Ange, Carlstead, unpublished], concentrations in Thailand were within the range, but mean concentrations were lower. The same EIA was employed, but the extraction technique differed, which might explain some of the differences. More work is needed to develop reference ranges for FGM, metabolic marker and lipid measures to determine what is ‘normal’ versus ‘abnormal’, although again, use of different laboratories and techniques can make interpretation challenging. This study also was only conducted for 1 year, so follow up observations are needed to determine if the patterns hold across years, and how changes in management influence subsequent results.

## Supporting information

S1 Table Individual fecal glucocorticoid metabolite (FGM) concentrations. Mean, median, minimum, maximum and SEM values are presented.
(XLSX)
